# Hierarchy is Detrimental for Human Cooperation

**DOI:** 10.1038/srep18634

**Published:** 2015-12-22

**Authors:** Katherine A. Cronin, Daniel J. Acheson, Penélope Hernández, Angel Sánchez

**Affiliations:** 1 Lincoln Park Zoo, Lester E. Fisher Center for the Study and Conservation of Great Apes, Chicago, IL 60614, USA; 2 Max Planck Institute for Psycholinguistics, Comparative Cognitive Anthropology Group, 6525 XD Nijmegen, The Netherlands; 3 Donders Institute for Brain, Cognition and Behaviour, Nijmegen, The Netherlands; 4 Universitat de València, ERI-CES, Departamento de Análisis Económico, Spain; 5 Universidad Carlos III de Madrid, Grupo Interdisciplinar de Sistemas Complejos (GISC), Departamento de Matemáticas, and Institute UC3M-BS of Financial Big Data, 28911 Leganés, Madrid, Spain; 6 Universidad de Zaragoza, Instituto de Biocomputación y Física de Sistemas Complejos (BIFI), 50018 Zaragoza, Spain

## Abstract

Studies of animal behavior consistently demonstrate that the social environment impacts cooperation, yet the effect of social dynamics has been largely excluded from studies of human cooperation. Here, we introduce a novel approach inspired by nonhuman primate research to address how social hierarchies impact human cooperation. Participants competed to earn hierarchy positions and then could cooperate with another individual in the hierarchy by investing in a common effort. Cooperation was achieved if the combined investments exceeded a threshold, and the higher ranked individual distributed the spoils unless control was contested by the partner. Compared to a condition lacking hierarchy, cooperation declined in the presence of a hierarchy due to a decrease in investment by lower ranked individuals. Furthermore, hierarchy was detrimental to cooperation regardless of whether it was earned or arbitrary. These findings mirror results from nonhuman primates and demonstrate that hierarchies are detrimental to cooperation. However, these results deviate from nonhuman primate findings by demonstrating that human behavior is responsive to changing hierarchical structures and suggests partnership dynamics that may improve cooperation. This work introduces a controlled way to investigate the social influences on human behavior, and demonstrates the evolutionary continuity of human behavior with other primate species.

Determining the conditions that facilitate cooperation in humans has been a challenge embraced by many disciplines; evolutionary biologists, psychologists, and social scientists have been attempting to tackle this question for decades^1^,^2^,^3^. Understanding when cooperation flourishes is of both theoretical and practical interest as our species faces environmental and societal challenges that may only be solved by working together^4^,^5^. Cooperation has been defined in many ways^6^; here we are referring to cases in which two or more individuals work together to achieve a common goal^7^. While this form of cooperation is not altruistic (nobody necessarily incurs a cost for cooperating), choosing with whom to cooperate and under what conditions to invest limited resources into cooperation poses a significant challenge for our species and others^8^,^9^,^10^.

The effect of the social environment on cooperation has received attention in studies of nonhuman animal behavior but has been largely overlooked in human research. Research with animals in the wild and under controlled conditions in captivity has consistently shown that social dynamics, and specifically the nature of the dominance hierarchy, has a large impact on cooperative outcomes^9^,^11^,^12^,^13^,^14^,^15^,^16^,^17^,^18^,^19^. Although variable in form, every animal society has some form of dominance hierarchy^20^,^21^. Hierarchy is defined as priority of access to resources and probability of winning competitive encounters^22^, and reflects underlying assymetries in power. A hierarchy can be characterized in terms of linearity and steepness^22^, with the former providing information about the degree of transitivity between individuals and the latter indicating the extent to which individuals differ from each other in winning encounters or accessing resources. Among nonhuman primates, it has been demonsrated repeatedly that the characteristics of dominance hierarchies impact cooperative outcomes, with steep and linear hierarchies being associated with decreased cooperation. For example, experiments have shown that cooperation is impeded among chimpanzees living in steep and linear hierarchies^16^,^23^, whereas it emerges more easily among species with more relaxed hierarchies such as cottontop tamarins^15^,^16^,^17^.

A great deal of research has focused on human cooperative behavior^24^, but these experiments have primarily been conducted with anonymous participants^25^,^26^, leaving the influence of social relationships on cooperation largely overlooked^27^. Although the influence of hierarchy on cooperation has rarely been examined, researchers have considered hierarchy’s influence on economic issues such as market entry^28^, bargaining^29^,^30^, and learning^31^. Other work has investigated how disproportionate power in sanctioning influences cooperation^32^, and both empirical^33^,^34^ and modeling^35^,^36^ investigations have been directed at how group status impacts cooperation and competition with other groups. In the current study, we hypothesize that social relationships, and specifically hierarchical relationships reflecting power assymetries between individuals, will have a negative impact on human cooperation as it does in our nonhuman primate relatives. In order to test the effect of social hierarchy on cooperation, we present participants with a task inspired by nonhuman primate research in which two individuals of known social rank are presented with the opportunity to invest in a cooperative task, and, if a threshold of investment is met and cooperation is achieved, the higher ranking of the two investors controls the distribution of the resource^16^,^37^,^38^. To investigate how human cooperation is impacted by the presence of a social hierarchy, we compare cooperative success in the presence of a hierarchy (with both earned and arbitrarily assigned ranks) to success in a condition when hierarchy is absent.

## Experimental design

In order to probe the effects of hierarchy on human cooperation, we adapted a classic mutualistic cooperation task employed in nonhuman primate studies^15^,^16^,^39^,^40^ for use with human participants. In this task, two individuals have the opportunity to work together to obtain some benefit that is not pre-divided for the players. In the present study, the only social feature we manipulated was hierarchy, which allowed us to isolate this effect while preventing confounding effects arising from other social relations. To generate the hierarchies, we asked groups of ten anonymous participants to carry out multiple unrelated tasks on a computer (see Methods Summary below and Supplemental Information for a full description), and ranked them according to their overall performance. Subsequently, participants were arranged in pairs and participants were informed of their own rank and the rank of the person with whom they were paired. In the *cooperation phase,* both players in the pair were assigned 20 units and given the option to contribute simultaneously any number of these units to a common pot, unaware of the partner’s contribution. ECUs not contributed to the common pot were not lost to the player but could be obtained in the final payout. If this pot reached or exceeded 20 units, then the pot doubled to 40 units for the *splitting phase*, otherwise they obtained nothing (i.e., their contributions to the pot were lost). Players were informed only whether the threshold was reached or not, and did not know *a posteriori* the amount contributed by their partner.

The splitting phase was implemented with an ultimatum game. The higher-ranked individual proposed how to split the 40-unit pot, and if the proposal was accepted by the lower-ranked person, each would receive the stipulated amount. If the lower-ranked person rejected the higher-ranked person’s proposal, s/he could attempt to compete and a lottery would assign the 40 units to one of the two members of the pair with probability proportional to their rank (which was known to the participants). Given that hierarchy is defined as priority of access to resources and the probability of winning competitive encounters^22^, these methods were designed to maintain the impact of hierarchy throughout the experiment.

Players played nine rounds, interacting with every other player in the group so all possible rank differences could be explored. Each player experienced one of three different treatments: the “earned hierarchy condition” (described above), a “random hierarchy condition” in which the ranking was assigned randomly without performing any task, or a control condition with no hierarchy. In the control condition, when the proponent’s offer was rejected, the whole pot was assigned to one of the players with equal probability.

## Results

In what follows, we analyze the experimental results using mixed-effects models, the appropriate technique when individuals are embedded within a group and are paired with each other across several interactions (and hence are not independent). This approach, combined with random selection of the individuals representing each dyad (when dyads were the proper level of analysis) and a bootstrap resampling technique, allows us to establish significance with a large degree of accuracy (see Supplemental Information).

**Figure 1:**
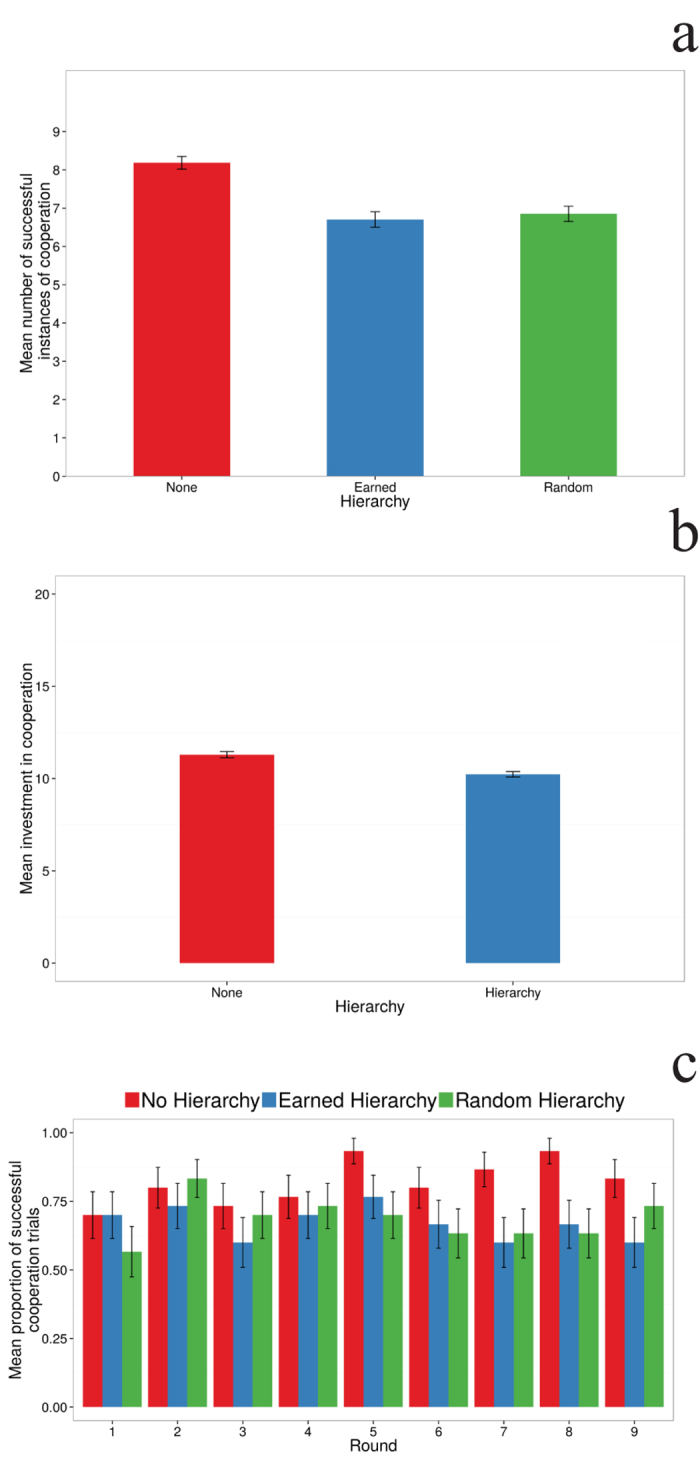
Success and contributions decrease in hierarchically organized groups, irrespective of the origin of the ranking. (**A**) The mean number of succesful instances of cooperation in the control condition in which there was no hierarchy was significantly higher than both hierarchical conditions. The maximum number of cooperative successes was 9. (**B**) The mean player contribution in the cooperative task with and without hierarchy. (**C**) Mean cooperation success as a function of round and hierarchy condition shows that there is a small but significant interaction among these variables, which might arise from the fact that the differences between the two treatments appear to increase as the experiment progresses. Colors correspond to the three types of hierarchical treatment as indicated in the plot.

First, we look at if (and how) the existence of a hierarchy affected success in the cooperation phase. Figure 1A shows that success (reaching or exceeding 20 units collectively) in the control condition was significantly more frequent than that of the hierarchically organized groups (95% CI for the coefficient, [0.14, 0.45]), indicating that participants were more prone to cooperation in the treatment lacking hierarchy. Interestingly, there was no difference between the random and earned hierarchy conditions, indicating that whether rank arose from personal performance or was randomly assigned did not affect cooperation success. Therefore, we pooled the two hierarchical treatments in subsequent analyses. In the control condition the average contribution to the pot was significantly greater than in the hierarchical treatments (95% CI for the coefficient, [0.23, 0.58]), cf. Fig. 1B) leading to more successful cooperation events in the absence of a hierarchy. We also found an interaction between the presence or absence of hierarchy and round of play, which emerged from the fact that the difference in cooperative success between the hierarchy and no hierarchy conditions increased as the experiment proceeded (95% CI for the coefficient, [0.007, 0.116], cf. Fig. 1C). Finally, we observed that there was a positive correlation between rank and total earnings in the experiment (see Supplemental Information), i.e., higher ranked participants received larger payments than lower ranked ones. Therefore, in this experiment we did in fact see that higher ranked individuals obtained more resources.

**Figure 2:**
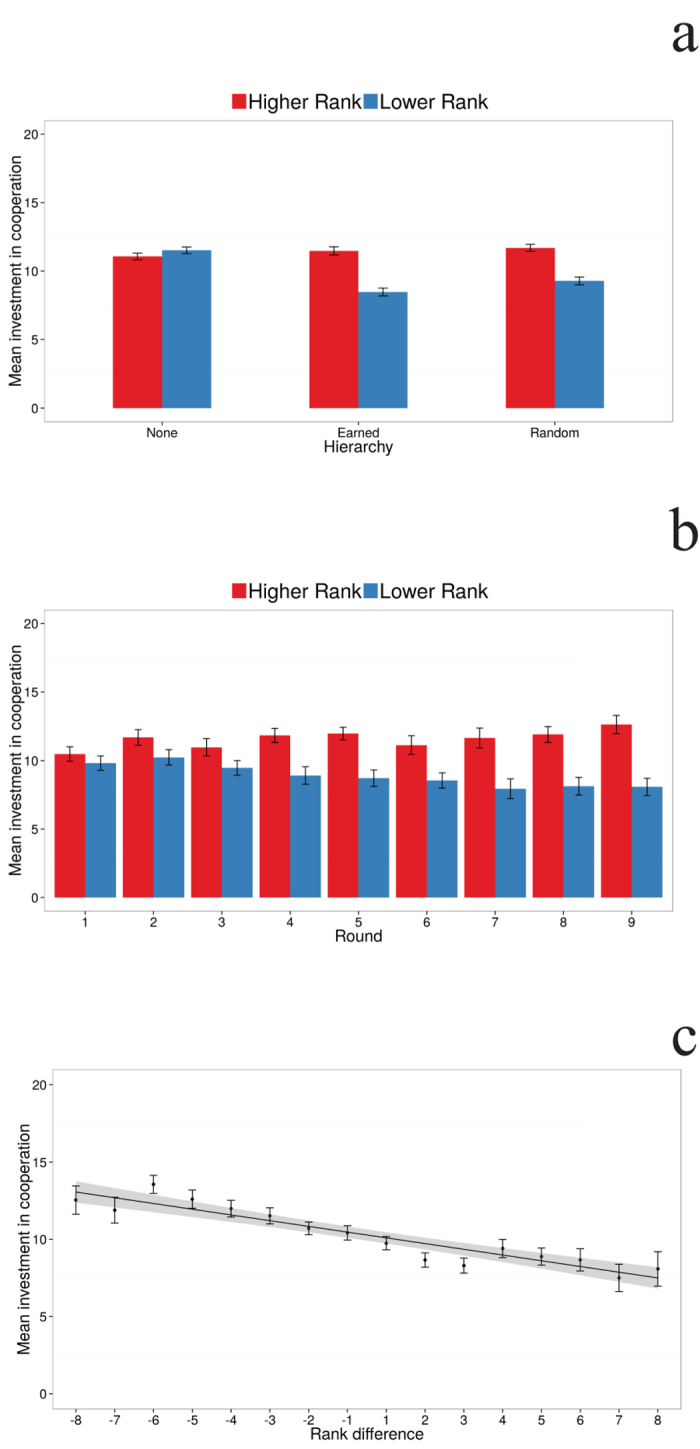
Contributions decrease in the hierarchy condition for lower ranking partners, and are predicted by the rank difference. (**A**) The contributions by the higher and lower ranked partners in the three experimental conditions, (**B**) the contributions by the higher and lower ranked partners in the hierarchy and non-hierarchy treatments across rounds, (**C**) the contribution as a function of the rank difference between the two partners of the dyad. Negative numbers correspond to higher ranking positions, e.g., −8 indicates the focal player (whose contribution we are evaluating), was ranked 1 and her partner was ranked 9).

**Figure 3:**
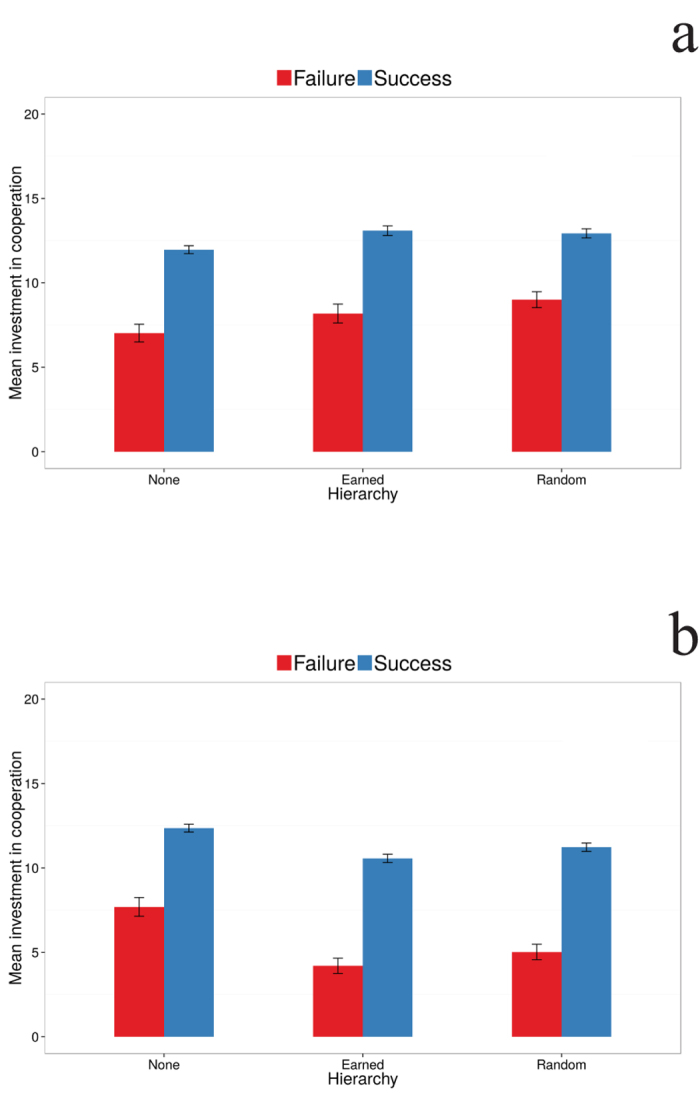
Contributions differ markedly in the cases when cooperation is or is not achieved. (**A**) Mean player contribution in the cooperative task when cooperation fails (red) and when it succeeds (blue) for the higher ranked player in the three experimental conditions. (**B**) The same for the lower ranked player. Note that when there is no hierarchy the behavior of both types of player is the same.

Why does cooperation suffer in the presence of a hierarchy? While the contributions from both partners are similar in the condition with no hierarchy, they differ clearly in the two hierarchical treatments (Fig. 2A). The main reason for the decrease in successful cooperation events can be traced to diminishing contributions by *lower* ranked individuals (Fig. 2B). Indeed, in this respect, our analysis shows that there is a significant difference in contributions between higher and lower ranked individuals (95% CI for the coefficient, [−1.65,−0.79]) and a significant interaction of rank and round (95% CI for the coefficient, [−0.30, −0.10]). Figure 2B shows that higher ranked participants increase their contributions as the experiment procedes while lower ranked participants decrease their contributions. Thus, it is the lack of contribution from the lower ranked players that leads to more cooperation failures. Figure 3 shows that, in the hierarchy conditions, lower ranking individuals contribute little in unsuccessful attempts (when the 20 unit minimum is not reached) compared to higher ranking individuals, and when cooperation is achieved, lower ranking individuals’ contributions barely surpass ten units. In view of this evidence, it appears that both individuals realized the consequences of their rankings on their potential earnings. Accordingly, lower ranked participants responded by reducing their investment, whereas higher ranked participants anticipated the reluctance of their partner and invested more in an attempt to rescue cooperation, but often not enough to be successful. In fact, when we examined whether the magnitude of the rank difference predicted cooperation investments, we found that the amount contributed by lower ranked participants increases (and the amount contributed by higher ranked participants decreases) as the rank difference became smaller, i.e., as the chances of receiving the whole pot by chance increased (95% CI for the coefficient, [−0.47, −0.25]) (cf. Fig. 2C).

**Figure 4:**
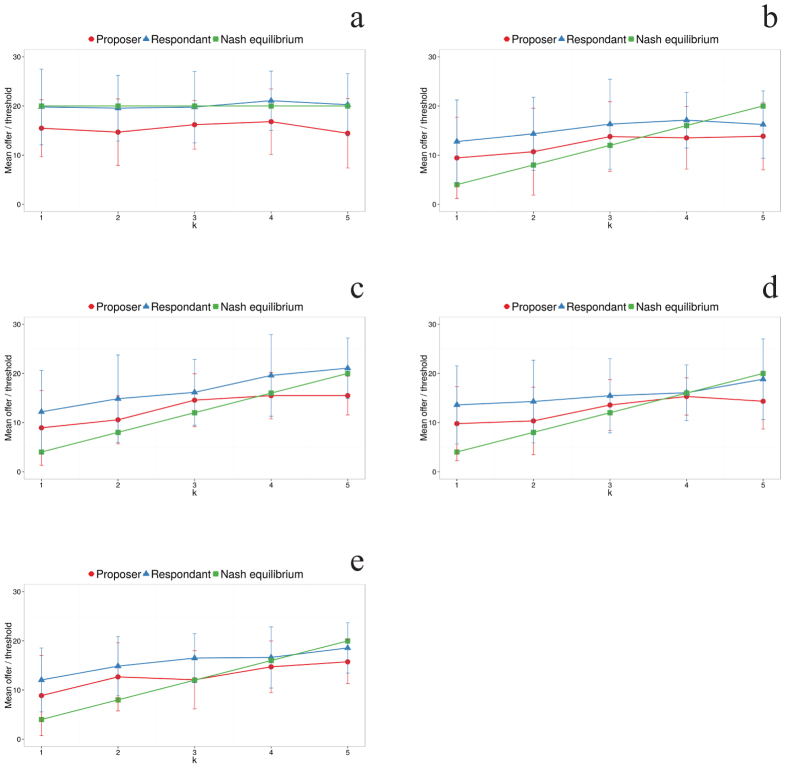
Offers and thresholds in the splitting phase behave qualitatively as predicted by Nash equilibrium. Mean offer and threshold in the ultimatum game with (hierarchy-based) outside option^41^ vs *k*, the groups of rank differences organized as indicated in the text, with rank difference being smaller with increasing *k*. Plots (**A**) through (**E**) correspond to our five treatments: (**A**) no hierarchy, full experiment (cooperation plus splitting); (**B**) earned hierarchy, no cooperation task; (**C**) random hierarchy, no cooperation task; (**D**) earned hierarchy, full experiment, and (**E**) random hierarchy, full experiment. In all plots, red circles correspond to the proposer’s offer, blue triangles correspond to the responder’s minimum acceptable offer, and the green solid line is the theoretical prediction of the Nash equilibrium for both the minimum acceptable offer and the proposer’s offer.

Let us now move on to the behavior of players in the ultimatum game with an (hierarchy-based) outside option^41^, or the “splitting phase”. In the absence of hierarchy, we observed that proponents offered on average 25% less than what receivers were willing to accept. When hierarchy was introduced, this difference was again observed, but proponents offered lower amounts for higher rank differences and receivers stated a lower minimum amount they would accept (Fig. 4). On the other hand, our analysis shows that both offers and expectations are independent of the investments made in the cooperation phase. This finding is further supported by the results of two additional treatments in which the cooperation phase was omitted and players proceeded directly from the hierarchy formation stage, be it earned or random, to the splitting phase. We did not observe any significant differences in the amounts offered and expected between these treatments and those in which there was a cooperation stage (see Supplementary Information). Such a result may be explained by a similar feeling of entitlement in the splitting phase regardless of whether this phase followed successful cooperation.

It is interesting to observe that, in the absence of hierarchy, i.e., when the chance to receive the pot is 0.5, respondents play very closely to the Nash equilibrium of the game, accepting only 20 units or more of the pot. This is much larger than minimum acceptable offers typically found in the standard ultimatum game^26^. This may have arisen because of the cooperation phase as discussed above. Alternatively, it seems more likely that this arises because respondents have a large chance to keep the whole pot when they refuse the offer in this experiment, contrary to the typical ultimatum game in which they would receive nothing. When hierarchy is present, Nash equilibrium predicts that the higher ranked individual should offer 4*k* (see “Subgame perfect equilibrium calculation” in the Supplementary Information), *k* being a measure of rank difference (*k* = 1 corresponds to a difference in rank of 8 or 9; *k* = 2, to 6 or 7; *k* = 3, to 4 or 5; *k* = 4, to 2 or 3, and *k* = 5, to a difference of 1), and the lower ranked individual should accept it. We do not presume that the participants make these calculations, rather, they adjust their behavior based on their understanding of the experiment and their experiences in previous rounds. In the present experiment, the behavior of the two partners is only qualitatively similar to the Nash equilibrium. Remarkably, higher ranked individuals make offers closer to the prediction, while lower ranked individuals expect to receive a significantly larger amount of the pot.

Another interesting observation is that for greater rank differences (lower *k*), the higher ranked individuals are prone to share an amount larger than the Nash prediction. Additionally, for all rank differences, receivers state a minimum amount that they would accept that is greater than theory and dictators’ action prescribe (cf. Fig. 4). This might arise from the fact that proponents perceive the splitting phase closer to a standard ultimatum game than it actually is. When their partner is much lower in rank, even if she rejects the offer, the proponent has a large probability to keep the whole pot (while in the ultimatum game the proponent would receive nothing). Accordingly, they offer a larger amount than the Nash equilibrium would predict, probably because they fear losing their share, to which they feel entitled. This agrees with the slopes of the rank dependence of offers and acceptance levels being lower than the Nash prediction; however, they are not completely horizontal as in the control condition because participants may still partially take into account the lack of refusal power of the respondent. Such consideration would effectively make the hierarchy less important, as the individuals in the lowest positions would be treated as if they were ranked higher. It is important to note, however, that the hierarchy itself is not changed, as it is fixed from the beginning of the experiment, and individuals with lower ranking were still offered less than intermediate-ranked ones.

## Discussion

We have shown that achieving cooperation among humans is more difficult when there is an underlying hierarchical structure producing different ranks between people and therefore unequal payoffs for the participants. This result is driven by insufficient contributions from lower ranked individuals who cannot be confident that they will benefit from cooperating. Remarkably, human behavior is consistent with a trend that permeates the rest of the primate order; primates in steeply hierarchical societies have difficulty cooperating for benefits that must be divided^16^,^21^,^42^, whereas primates organized in weakly hierarchical (egalitarian) societies are more successful^16^,^37^. Whether this pattern holds outside of the primate order requires additional research given that dyadic cooperative challenges have only rarely been presented to other taxa (but see refs 43, 44, 45, 46, 47 for related studies on dogs, hyenas, rooks, parrots and elephants). Hierarchies reliably predict resource distribution in nonhuman primates, therefore attempts to complete a joint task are presumably hampered by the lower ranked individual’s awareness that the higher ranked individual will keep the whole pot for itself. For example, among one of our closest living relatives, chimpanzees (*Pan troglodytes*), lower ranked individuals avoided engaging with higher ranked individuals in a task producing rewards that must be divided^48^ whereas egalitarian cottontop tamarins (*Saguinus oedipus*) readily perform a joint task for undivided rewards^37^. The same impact of hierarchy appears to be true in our own species, suggesting continuity in the evolution of mutualistic cooperation among humans and other primates.

This study was designed to recreate key aspects of cooperation studies conducted with nonhuman primates to enable comparisons across species. However, one notable difference between the present study and studies conducted with nonhuman species is the inclusion of verbal instructions for participants and the opportunity for participants to ask clarifying questions. As comparative approaches to studying the evolution of economic behavior continue to develop, it would be beneficial to develop methods that do not require explicit instruction to human participants and rely more heavily on participants’ learning the boundaries of the games through experience^49^.

As our experiment was designed with a small number of rounds to avoid the reputation effects that may result from playing twice with the same partner, we are prevented from examining whether learning or previous experience affects individual behavioral sequences. As mentioned above, the data suggest that participants’ cooperation changes over rounds, but the corresponding effect is small. Given a longer sequence of repeated interactions, it might be possible that members of a hierarchically organized group would partially overcome mistrust of higher ranked individuals in those that establish generous reputations^50^ and these individuals might be able to elicit more contributions from their lower ranked partners. Interestingly, this could be the reason why in early societies individuals achieved a degree of preeminence by organizing parties or celebrations for the rest of the tribe or group^51^. However, it is clear that such a mechanism is not an easy one to implement and it does not arise spontaneously; in fact, in many early societies the ‘generosity path’ to becoming influential is often highly regulated, consisting of a set of increasingly complex and expensive parties one has to throw to advance in rank^51^. In any event, the outcome of our experiment suggests specific points on which group members could be encouraged to take action so cooperation is increased and points to the detrimental effects of even transient, arbitrary hierarchies. Moving forward, experiments in artificial social contexts like ours appear to be a very powerful tool to examine strategic behavior in socially relevant situations. We hope that our work will stimulate further work along these lines.

## Methods

### Experimental setup

The experiment was carried out with volunteers chosen among the LINEEX subject pool. All participants in the experiments reported in the manuscript signed an informed consent to participate. In agreement with the Spanish Law for Personal Data Protection, their anonymity was always preserved. This procedure was approved by the Vice Provost of Research of Universidad Carlos III de Madrid, the institution responsible for funding the experiment, and the experiment was subsequently carried out in accordance with the approved guidelines.

Experiments corresponding to different groups were scheduled at different dates, namely March, 5th and 6th and April, 3rd 2014. Participants played through a web interface specifically designed for the experiment (see below) accessible from the computers of the lab. At least three researchers supervised the experiment in the room (which had a maximum capacity of 64 players), preventing any interaction among the participants. They were not allowed to talk or signal in any way. In order to further guarantee that potential interactions among players seated next to each other in the room did not influence results, assignment to computer stations was random and stations were isolated from each other by opaque panels. Hence there was no relationship between physical proximity and interactions in the game.

All participants were provided with printed instructions (see Supplementary Information). When everybody had read the instructions, a supervisor read them aloud and verbally confirmed that everybody had understood the rules of the game. Any question was privately resolved via one on one interactions between an experimenter and a participant. Once all questions had been answered, the first phase of the experiment began.

240 participants (140 male, 100 female) between ages 18 and 55 took part in the experiment. 180 of them (113 male, 67 female) took part in treatments that required cooperation to achieve the pot; the remainder proceeded directly to the splitting phase. All participants were in the same room as the rest of their group and informed that they were playing real people. In treatments where hierarchy was earned, participants were asked to carry out computer tasks in order to assign ranks within groups consisting of 10 participants. Participants were presented with a quiz similar to other economic experimentss^29^,^30^,^31^, but were also asked them to play Tetris and to carry out arithmetic operations. The total number of points accumulated in the three tasks was used to rank the participants.

Participants remained at their computer station to perform each task, but were sequentially paired with all other participants in their group in a random order for the cooperative task. Prior to each round, each participant was provided with the rank of their partner. In the cooperative task, participants were given the opportunity to donate to a pot in order to surpass a 20 unit threshold. Participants were informed whether or not they met the 20 unit threshold for successful cooperation but were unaware of the contributions of their partners. If the threshold was met, the pot was doubled to 40 units and the splitting phase was carried out using an ultimatum game with an (hierarchy-based) outside option^41^. If the split was refused by the lower ranking partner, s/he could choose to compete and a lottery would assign the whole pot to one of the two members of the pair with probability proportional to their rank. At the end of the experiment, units were exchanged at a rate of 15 units  =  1 euro. Total earnings for the games of the experiment ranged from 13.00 to 36.00 euros.

Utility theory predicts that individuals should behave in the way that maximizes their own gains (for this experiment, see Supplemental Information for the subgame perfect equilibrium calculation). However, it is worth noting that if players were to deviate from rational choice and instead employ a strategy that minimizes inequity between players (e.g., ref 52), lower ranking players may gain some additional control and achieve equality at a personal loss by refusing to invest in phase 1 or by raising their acceptance threshold in phase 2. This is not equivalent to a role reversal given the definition of hierarchy above because lower ranked players will not gain more than their higher ranking partner, but it may reduce the difference in gains. Interestingly, even under this alternative interpretation, the conclusion does not differ in that the presence of the hierarchy destabilizes cooperative interactions.

### Statistical analysis

All statistical analyses were conducted using R versión 3.10^53^, with mixed-effects models calculated using the lme4 package^54^. The analysis of cooperation success was conducted using a linear mixed-effects model with a logistic linking function, where success was coded as 1 and failure as 0. Round was mean-centered prior to analysis, whereas the effects of hierarchy were contrast coded. The first contrast compared the effect of no hierarchy (+2) to the hierarchy conditions (random and earned coded as −1 and 1 respectively). The second contrast compared random (−1) vs earned (+1) hierarchy. As no effect of random vs. earned was observed in this analysis, only the contrast between the no-hierarchy and hierachy conditions was included in subsequent analyses.

As cooperation success was a function of the actions of both individuals, dyad was treated as the grouping variable to estimate random intercepts and a random slope for round. Parameters were estimated using a bootstrap procedure in which one individual of each dyad was randomly selected without replacement. 100 bootstrap samples were drawn for this and all subsequent analyses, and 95% confidence intervals were extracted from the bootstrapped estimates of the parameters. The analysis of the amount of money contributed to the cooperation task was conducted in a similar fashion, although in this instance, the amount contributed was treated as a continuous variable, and random intercepts and slopes for round were estimated for the individual.

The analysis of mean cooperation offer as a function of round and rank was conducted in two ways. First, rank was treated as a binary variable, coded as +1 for higher rank and −1 lower rank. This analysis was conducted relative to the mean offer in the no hierarchy condition. A random intercept and random slopes for each variable were estimated using the individual as the grouping variable. A second analysis treated rank difference continuously. The highest and lowest ranked individuals were excluded from this analysis as no random effects could be estimated for these individuals (they were always the highest/lowest ranked).

The analysis of the ultimatum offers and expected offers compared the effects of the presence/absence of a cooperation round and rank difference. Here, the presence of a cooperation round was coded as +1, and the absence as -1. Role was coded as +1 for the person receiving, and −1 for the person offering. The absolute value of the rank difference was also included, thus a difference of +/−1 was coded as 1, +/−2 as 2, etc. This allowed us to assess the relative effect of rank difference on ultimatum offers/expectations regardless of the direction of the difference. Mean-centered round was also included, as well as estimates of the random intercept and random slopes for round and rank difference using individuals as the grouping variable.

## Additional Information

**How to cite this article**: Cronin, K. A. *et al.* Hierarchy is Detrimental for Human Cooperation. *Sci. Rep.* **5**, 18634; doi: 10.1038/srep18634 (2015).

## Supplementary Material

Supplementary Information
