# Supplementary material for: Hierarchy is Detrimental for Human Cooperation
Source: Sci Rep. 2015 Dec 22;5:18634. doi: 10.1038/srep18634 (PMC5155906; doi:10.1038/srep18634)
Supplement: Supplementary Information [file srep18634-s1.pdf]

Supplementary Information
Hierarchy is detrimental for human cooperation

Katherine A. Cronin[1,2], Daniel J. Acheson[3], Penélope Hernández[4], Angel Sánchez[5,6]

[1] Lincoln Park Zoo, Lester E. Fisher Center for the Study and Conservation of Great Apes, Chicago, IL 60614, USA

[2]Comparative Cognitive Anthropology Group, Max Planck Institute for Psycholinguistics, Wundtlaan 1, 6525 XD Nijmegen, The Netherlands

[3]Donders Institute for Brain, Cognition and Behaviour, Nijmegen, The Netherlands

[4]ERI-CES, Departamento de Análisis Económico, Universitat de València, Spain

[5]Universidad Carlos III de Madrid, Grupo Interdisciplinar de Sistemas Complejos (GISC), Departamento de Matemáticas, and Institute UC3M-BS of Financial Big Data, 28911 Leganés, Madrid, Spain

[6]Instituto de Biocomputación y Física de Sistemas Complejos (BIFI), Universidad de Zaragoza, 50018 Zaragoza, Spain

1. Experimental instructions

Since there were several treatments (no hierarchy, earned hierarchy, random hierarchy, and presence or absence of a cooperation phase), there were several different sets of instructions. A translation of the original Spanish instructions for the treatment with cooperation and earned hierarchy is provided below, indicating the place and alternative texts for the other treatments. All sets of instructions are available upon request.

English translation of the instructions

The purpose of this experiment is to study how individuals make decisions in certain contexts. The instructions are simple and if you follow them carefully you will receive cash at the end of the experiment in a confidential manner. You can ask questions you may have at any time by raising your hand. Aside from these questions, any communication is prohibited and subject to immediate exclusion from the experiment.

INSTRUCTIONS:

In this experiment sections of 10 participants are formed randomly. You will remain in the same section throughout the entire experiment. This experiment has two phases.

In the first phase each you will carry out individual tasks for 3 minutes each.

1. In the first task, you will execute additions with three two-digit numbers. Each correct addition will give you one point.
2. In the second task, you will play the game Tetris. For each deleted row, you will obtain one point.
3. In the third task you will answer general culture questions in a quiz format.,For each question you will be provided with four possible answers. Only one of them is correct. Each correct answer will give you one point.
4. Finally, you will be able to choose one of these tasks in order to obtain additional points..

All of the points you obtain will be summed up. The more points you obtain, the better positioned you will be for the second phase of the experiment.

[*In the control (no hierarchy) condition, the text starting with* "In the first phase" *and ending with* "second phase of the experiment" *was suppressed.*]

[*In the random hierarchy condition, the text for the first phase was simply:* "Each of you is assigned a random score from other sessions. The person with the highest score will be in 1st place, the second will be the 2nd, and so on, with the person with the lowest score achieving 10th place."]

The second phase has 9 rounds. In each round you will be paired with one of the members of your section, depending on his score and yours. Therefore, in each round, 5 pairs in each round will be formed from the section of 10 people. Your pairings will depend on the difference between your score and your partner's.

[*In the control (no hierarchy) condition, the last sentence was suppressed.*]

In each round, the player with the highest score will be Player A; the other player will be Player B.

At the beginning of each round, you will be provided the following information:

- The score of every section member, including your own score (in green) and your partner's (in grey).
- A table of ranks for everyone in your section, yours will be marked in green.

The pair with the greatest difference between their scores (or ranks) will be called group 1. The pair with the second highest difference will be group 2, and so on. In each round you will see if your score is higher or lower than your partner's.

Each of players has 20 ECUs (experimental virtual currency) as an initial budget for each of the 9 rounds to play in the second phase. In each round, each player may make a contribution between 0 and 20 ECUs. If the sum of the two contributions is greater or equal than 20 ECU, then you will access 40 ECUs for the two members. If it is less than 20 ECUs, then nothing will be distributed.

[*When the treatment did not have a cooperation phase, the above paragraph was substituted by the following sentence:* "In the next phase you will have to decide on how to split 40 ECUs".]

How will the ECUs be shared?

Player A in each pair can share 40 ECUs. S/He decides how many of the 40 ECUs s/he gives to player B (r_A).

Player B will indicate the least amount of ECUs s/he accepts from Player A (r_B).

If this amount is less or equal than the one given from Player A, $r_B \leq r_A$, then the share is accepted by Player B, and each player will obtain the division share decided by Player A (r_A). The profit of Player A: 40 - r_A; the profit of Player B: r_A.

In the opposite case $r_B > r_A$, the share will not be accepted by player B. Then, the computer will randomly choose a number from 1,2,…,10. If your group is x, and the number chosen by the computer is lower or equal than 10-x, then Player A will earn the total 40 ECUs. Otherwise, if the computer chooses a number greater than 10-x, Player B will earn the total 40 ECUs.

[*In the control (no hierarchy) condition, the text starting with* "If your group is x" *and ending with* "the total 40 ECUs" *was substituted by the following one:* "If the number chosen by the computer is

lower or equal than 5, then Player A will earn the total 40 ECUs. Otherwise, if the computer chooses a number larger than 5, Player B will earn the total 40 ECUs".]

What is the profit at the end of the round?
The ECUs that you do not invest from your original 20 ECUs plus the sharing profit, if any.

Your total profit will be the sum of the profit from each round. At the end of the experiment, your total profit in ECUs will be changed to EUROS at the following rate of exchange: 15 ECUs = 1 Euro and you will receive this amount of cash after you complete a short questionnaire.

2. Statistical Results: Parameter estimates for all analyses of Cooperation Success, Cooperation Offer and Ultimatum Offers

2.1. Probability of Success by Hierarchy Condition and Round

		95% CI		
	Estimate	Lower	Upper	Random Effects
Intercept	1.09	0.87	1.31	dyad
Round	0.04	-0.04	0.12	dyad
Hierarchy vs. No Hierarchy	0.29	0.14	0.45	
Earned vs. Random Hierarchy	-0.04	-0.27	0.20	
Hier.v.No Hier X Round	0.06	0.01	0.12	
Earned.v.Random X Round	-0.02	-0.10	0.06	

Generalized linear mixed effects model with a logistic linking function. Parameter estimates correspond to logits. Dyads (partners) were included as a random effect. Round was centered, and Hierarchy vs. No Hierarchy and Earned vs. Random and were contrast coded. Results show a main effect of Hierarchy vs. No Hierarchy, and a Hierarchy vs. No Hierarchy X Round interaction.

2.2. Cooperation offer as a function of Hierarchy Condition and Round

		95% CI		
	**Estimate**	**Lower**	**Upper**	**Random Effects**
Intercept	10.63	10.37	10.89	
Round	-0.01	-0.10	0.09	subject
Hierarchy vs. No Hierarchy	0.40	0.23	0.58	
Hier.vs.noHier X Round	0.01	-0.04	0.05	

Generalized linear mixed effects model. Random effects were estimated within subjects. Round was centered, and Hierarchy vs. No Hierarchy. Earned and random hierarchy were collapsed. Results show only a main effect of Hierarchy vs. No Hierarchy.

2.3. Cooperation offer as a function of Binary Rank and Round

		95% CI		
	Estimate	Lower	Upper	Random Effects
Intercept	-1.06	-1.49	-0.63	subject
Round	-0.05	-0.18	0.07	subject
Rank (Binary)	-1.22	-1.65	-0.79	subject
Rank X Round	-0.20	-0.30	-0.10	subject

Generalized linear mixed effects model. Random effects were estimated within subjects. Round was centered, and Rank was coded with higher ranked individuals coded as +1 and lower ranked as -1. Results are presented relative to the mean of the no hierarchy condition. Thus, the negative intercept indicates the lower offers for the hierarchy relative to the no hierarchy condition. Results show a main effect of Rank and a Rank X Round interaction.

2.4. Cooperation offer as a function of Cooperation Round Present, Absolute Rank Difference, Role and Round in the Ultimatum Game

		95% CI		
	Estimate	Lower	Upper	Random Effects
Intercept	16.85	15.85	17.85	
Cooperation Round Present	0.05	-0.86	0.96	
Round	-0.39	-0.59	-0.20	subject
Role	-1.52	-2.21	-0.83	
Rank Difference	-0.70	-0.94	-0.46	subject
Cooperation Present X Round	-0.14	-0.33	0.04	
Cooperation Present X Role	0.38	-0.37	1.14	
Round X Role	-0.06	-0.20	0.09	
Cooperation Present X Rank Difference	-0.12	-0.34	0.11	
Role X Rank Difference	-0.06	-0.28	0.17	
Cooperation Present X Round X Role	-0.06	-0.18	0.06	
Cooperation Present X Role X Rank Difference	-0.11	-0.31	0.10	

Generalized linear mixed effects model. Random effects were estimated within subjects. Round was centered, Cooperation Round Present as +1 present, -1 absent, and Role as coded with offers coded as -1 and expected coded as +1. Rank difference was coded as the absolute value of the rank difference. Results show a main effect of Round, Role and Rank Difference. Participants offered less as the rounds increased and offered more if they were higher ranking.

2.5. Total earnings as a function of rank

	Estimate	SE	t-value	p-value
Intercept	196.5	8.25		
Rank	-9.75	1.33	-7.33	<0.001

Linear regression of the effect of rank on total earnings. Earnings were pooled across all rounds for each participant, and a standard linear regression was run predicting those earnings as a function of rank, where lower values correspond to higher rank. Results show that higher ranked individuals earned more overall, with a 1 unit change in rank associated with 9.75 more ECUs.

3. Subgame perfect equilibrium calculation

The Subgame perfect equilibrium of the game can be calculated as follows. We start from the fact that the game has three stages, and depends on *k,* the difference in rank between the two participants. Therefore we proceed by backward induction to compute the Nash equilibrium at each stage starting with the last stage.

Let us describe the sequential game: In stage 1, players have to choose simultaneously their level of contribution to the common pot. Let s_1 and s_2 be the contributions of the higher- and lower-ranked player, respectively. Both quantities must be positive and less than the initial 20 units. If the players succeed in gathering 20 units among the two contributions, they proceed to the splitting phase. Player 1, the higher-ranked one, offers an amount x_1, between 0 and 40 units (the total pot to be shared) that is subsequently accepted or rejected by player 2, the lower-ranked one. In case of rejection, one of the players will be awarded the whole pot with probability proportional to their rank difference. In view of the fact that the expected payoff for player 2 case of rejection is $W_2=4k$, player 2 should reject if offered a smaller amount. On the other hand, the expected payoff for player 1 in case of rejection is $W_1=4(10-k)$, so she must at least secure that amount with her offer. This leads to a prediction for the splitting phase that player 1 should offer $4k$ and this should be accepted. Regarding the cooperation phase, it is clear that there is a multiplicity of equilibria, as any s_1, s_2 such that $s_1 + s_2$ is at least 20 gives access to the second phase, that would lead to a non-negative additional contribution to the payoff. In any event, the most relevant conclusion for our analysis in the main text is that the offer is predicted to be $4k$, and so should the acceptance threshold be.
